# Migratory behaviours are risk-sensitive to physiological state in an elevational migrant

**DOI:** 10.1093/conphys/coae029

**Published:** 2024-05-17

**Authors:** Kristin Denryter, Thomas R. Stephenson, Kevin L. Monteith

**Affiliations:** Haub School of Environment and Natural Resources, University of Wyoming, Bim Kendall House 804 E Fremont St, Laramie, WY 82072, USA; California Department of Fish and Wildlife, Sierra Nevada Bighorn Sheep Recovery Program, 787 N Main St., Bishop, CA 93514, USA; Haub School of Environment and Natural Resources, Wyoming Cooperative Fish and Wildlife Research Unit, Department of Zoology and Physiology, University of Wyoming, Bim Kendall House 804 E Fremont St, Laramie, WY 82072, USA

**Keywords:** body condition, facultative migration, ingesta-free body fat, migratory portfolio, nutritional buffer, ram, trade-off

## Abstract

Accretion of body fat by animals is an important physiological adaptation that may underpin seasonal behaviours, especially where it modulates risk associated with a particular behaviour. Using movement data from male Sierra Nevada bighorn sheep (*Ovis canadensis sierrae*), we tested the hypothesis that migratory behaviours were risk-sensitive to physiological state (indexed by body fat). Sierra bighorn face severe winter conditions at high elevations and higher predation risk at lower elevations. Given that large body fat stores ameliorate starvation risk, we predicted that having small body fat stores would force animals to migrate to lower elevations with more abundant food supplies. We also predicted that body fat stores would influence how far animals migrate, with the skinniest animals migrating the furthest down in elevation (to access the most abundant food supplies at that time of year). Lastly, we predicted that population-level rates of switching between migratory tactics would be inversely related to body fat levels because as body fat levels decrease, animals exhibiting migratory plasticity should modulate their risk of starvation by switching migratory tactics. Consistent with our predictions, probability of migration and elevational distance migrated increased with decreasing body fat, but effects differed amongst metapopulations. Population-level switching rates also were inversely related to population-level measures of body fat prior to migration. Collectively, our findings suggest migration was risk-sensitive to physiological state, and failure to accrete adequate fat may force animals to make trade-offs between starvation and predation risk. In complex seasonal environments, risk-sensitive migration yields a layer of flexibility that should aid long-term persistence of animals that can best modulate their risk by attuning behaviour to physiological state.

## Teaser

Interactions between physiological and behavioural adaptations to seasonal environments may be key to unravelling the mystery of partial migration. Physiological state, as indicated by body fat, influenced probability of migration in bighorn sheep as well as migration distance and probability of switching migratory tactics across years.

## Graphical Abstract

**Graphical Abstract:**
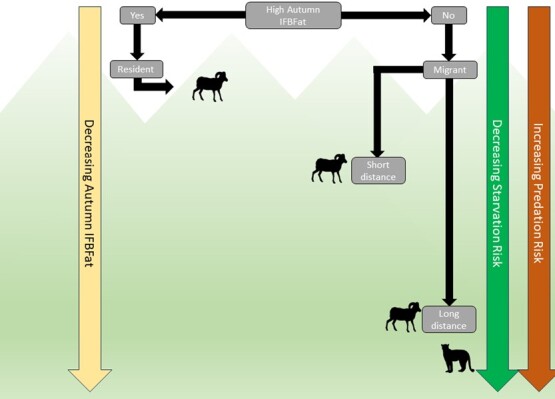

## Introduction

In highly seasonal environments, animals have evolved a suite of adaptations to cope with dramatic changes in the amount, quality and distribution of available resources between seasons. Physiological adaptations, such as circannual and circadian rhythms ([Bibr ref23][Bibr ref23][Bibr ref23]; Loudon,^1989^; [Bibr ref32][Bibr ref32][Bibr ref32]; [Bibr ref4][Bibr ref4][Bibr ref4]), seasonality of metabolic rates ([Bibr ref14][Bibr ref14][Bibr ref14][Bibr ref14]; [Bibr ref56][Bibr ref56][Bibr ref56][Bibr ref56]), seasonal and physiological changes in gut morphology and volume ([Bibr ref67][Bibr ref67][Bibr ref67]) and seasonal hypo- or hyperphagia ([Bibr ref47][Bibr ref47][Bibr ref47]; [Bibr ref6][Bibr ref6][Bibr ref6]; [Bibr ref26][Bibr ref26][Bibr ref26]) contribute to changes in food acquisition and nutrient assimilation that underpin an annual cycle of body mass and fat (Mautz,^1978^; [Bibr ref16][Bibr ref16][Bibr ref16]; [Bibr ref5][Bibr ref5][Bibr ref5][Bibr ref5]; [Bibr ref63][Bibr ref63][Bibr ref63]; [Bibr ref55][Bibr ref55][Bibr ref55]). Body fat stores are particularly significant to large-bodied endotherms that rely on these to finance survival and reproduction in seasonal environments ([Bibr ref16][Bibr ref16][Bibr ref16]; [Bibr ref5][Bibr ref5][Bibr ref5][Bibr ref5]; [Bibr ref44][Bibr ref44][Bibr ref44]; [Bibr ref63][Bibr ref63][Bibr ref63]; [Bibr ref55][Bibr ref55][Bibr ref55]). The importance of body fat in seasonal environments may extend beyond direct effects on survival and reproduction, particularly where the relative value of body fat as a nutritional buffer varies across behavioural states ([Bibr ref19][Bibr ref19][Bibr ref19]), but this notion remains largely untested.

According to the risk-sensitivity hypothesis, animals should aim to optimize resource gains (or fitness) relative to constraints via two possible strategies: risk-averse and risk-prone. Classically, risk-averse strategies were characterized by behaviours that result in a consistent, but modest reward, so there is little risk of not being rewarded ([Bibr ref12][Bibr ref12][Bibr ref12]). In contrast, risk-prone strategies were characterized by behaviours that result in a variable reward value that can change and be more valuable or less valuable than the constant reward ([Bibr ref12][Bibr ref12][Bibr ref12]). In risk-prone strategies, animals risk the constant (guaranteed) reward for the possibility of gaining a greater pay-off; the pay-off, however, may not be realized. Although classical work explored risk sensitivity relative to exogenous resources ([Bibr ref12][Bibr ref12][Bibr ref12]; Stephens,^1980^), more recent work has demonstrated risk sensitivity relative to endogenous resources also occurs. For example, female ungulates alter investment in survival and reproduction relative to body fat stores in a risk-sensitive (averse) manner ([Bibr ref7][Bibr ref7][Bibr ref7]; [Bibr ref44][Bibr ref44][Bibr ref44]; [Bibr ref55][Bibr ref55][Bibr ref55]). For animals that rely on accretion of body fat to finance life in seasonal environments, risk sensitivity relative to body fat may be critical to determining the adaptive value of seasonal behaviours including migration ([Bibr ref55][Bibr ref55][Bibr ref55]).

Migration occurs almost universally amongst vagile species, but is as diverse and complex as it is common, and comprises decisions at multiple spatial and temporal scales ([Bibr ref1][Bibr ref1][Bibr ref1]). At coarse scales, animals choose a migratory tactic, whereas at finer scales, animals choose when to migrate, where to migrate (or how far to move) and how long to remain on their migratory range. Traditionally, migratory tactics were considered dichotomous—either animals migrated (i.e. made a single round-trip movement between two distinct ranges) or they were residents that remained on a single range year-round. We now understand migration and residency are ends of a continuum that include intermediate migratory tactics such as making multiple round trips between ranges (e.g. ‘vacillators’ or ‘commuters’) or using more than two ranges (e.g. ‘mixed migrants’ or ‘multi-range migrants’) ([Bibr ref9][Bibr ref9][Bibr ref9]; [Bibr ref11][Bibr ref11][Bibr ref11]; [Bibr ref21][Bibr ref21][Bibr ref21]; [Bibr ref37][Bibr ref37][Bibr ref37][Bibr ref37][Bibr ref37]; [Bibr ref22][Bibr ref22][Bibr ref22]). Some individuals and populations employ the same tactic year after year, exhibiting little migratory plasticity ([Bibr ref52][Bibr ref52][Bibr ref52]), whereas others have more mutable (plastic) migratory behaviours and choose different tactics across years ([Bibr ref60][Bibr ref60][Bibr ref60]). Why some individuals and populations exhibit greater diversity in migratory tactics than others remains enigmatic, but understanding the relationship between migratory portfolios and demography may be important to conservation and management ([Bibr ref39][Bibr ref39][Bibr ref39]). Risk-sensitive decision-making relative to body fat, however, could be a plausible explanation because the relative value of body fat to survival differs amongst migratory tactics ([Bibr ref19][Bibr ref19][Bibr ref19]) and because the choice between residency and migration may itself reflect a trade-off between nutrition and predation.

In a risk-sensitive paradigm of migration for partially migratory populations, animals should choose a migratory tactic that affords them the greatest potential fitness pay-off. Where large quantities of body fat are critical for survival of individuals pursuing a given migratory tactic, animals should only choose that tactic if they have achieved adequate body fat stores ([Bibr ref18][Bibr ref18][Bibr ref18]; [Bibr ref19][Bibr ref19][Bibr ref19]). If an individual does not achieve adequate body fat stores for that migratory tactic, they should choose an alternative. For example, in altitudinal or elevational migrants, residents remain at high elevations during winter, where food supplies are depauperate compared with lower elevations, but predators are generally less common ([Bibr ref36][Bibr ref36][Bibr ref36]; [Bibr ref30][Bibr ref30][Bibr ref30]; [Bibr ref59][Bibr ref59][Bibr ref59]; [Bibr ref28][Bibr ref28][Bibr ref28]). For those residents to survive winter, they rely strongly on body fat stores ([Bibr ref19][Bibr ref19][Bibr ref19]) and therefore, high levels of body fat should be a prerequisite for residency in a risk-sensitive framework of migration. In contrast, migrants that move to lower elevation ranges with less snow and more abundant food supplies should be less reliant on body fat for overwinter survival, but also face greater risk of predation ([Bibr ref36][Bibr ref36][Bibr ref36]; [Bibr ref30][Bibr ref30][Bibr ref30]; [Bibr ref59][Bibr ref59][Bibr ref59]; [Bibr ref28][Bibr ref28][Bibr ref28]). If choice of migratory tactic is risk-sensitive to body fat, we predict that migratory propensity would increase with decreasing body fat stores. Essentially, body fat would allow residents to exhibit a risk-prone strategy to starvation, whilst being risk-averse to predation. In contrast, migrants would have lower body fat levels and be risk-averse to starvation (i.e. selecting a strategy with a more consistent food reward), but simultaneously risk-prone to predation. Further, animals may attune their migratory decisions at finer temporal and spatial scales relative to body fat to further reduce their risks. For example, body fat levels may dictate how far down in elevation an individual moves because predation risk is highest on low-elevation ranges ([Bibr ref35][Bibr ref35][Bibr ref35]) and hence animals should stay as high in elevation as their body fat stores would allow (i.e. risk-averse to starvation, but risk-prone to predation).

To test the hypothesis that choice of migratory behaviour was risk-sensitive to physiological state (as indicated by body fat), we used movement and body fat data from a migratory ungulate with high levels of migratory plasticity—Sierra Nevada bighorn sheep (*Ovis canadensis sierrae*; hereafter Sierra bighorn). In a risk-sensitive model of migration, differences in body fat also could underpin facultative migration and migratory plasticity. Hence, we also hypothesized that differences in body fat underpin facultative migration and predicted that at the population level, switching rates would increase with decreasing body fat because as body fat levels decrease, animals exhibiting migratory plasticity should modulate their risk of starvation by switching migratory tactics. We also assumed that decreasing body fat levels would require animals to be more plastic because at higher levels of body fat, they have a buffer against environmental conditions, but at lower levels of body fat, they lack that buffer and need to be more responsive to the immediate environment. Sierra bighorn face severe winter conditions at high elevations and higher predation risk at lower elevations.

## Materials and Methods

### Study area

Sierra bighorn live in 14 subpopulations in four recovery units (RUs; Central, Kern, Northern, Southern; i.e. metapopulations) throughout the Sierra Nevada Mountain Range (hereafter Sierra Nevada) in California. Bighorn sheep are highly gregarious, occurring in small bands that are sexually segregated for much of the year in groups of females and lambs (but sometimes include young males up to ~2 years old) and bachelor groups of older males ([Bibr ref8][Bibr ref8][Bibr ref8]; [Bibr ref53][Bibr ref53][Bibr ref53]). The eastern Sierra Nevada is in a rain shadow that supports xeric vegetation communities, including Great Basin sagebrush-bitterbrush scrub communities at low elevations (1500–2500 m); pinyon-juniper woodlands, subalpine meadows and subalpine forests at mid-elevations (2500–3300 m); and sparsely vegetated areas and alpine meadows at high elevations (>3300 m) ([Bibr ref36][Bibr ref36][Bibr ref36]). Summers in the Sierra Nevada are warm and dry; snow is the primary source of precipitation, with winter (Oct–Apr) snow depths ranging from 0 to 3.5 m and 0.4–14.9 m in southern and northern portions of the study area during 2006–19 (Aspendell-Bishop and Lee Vining, CA, respectively; National Weather Service, accessed 25 December 2022). Strong winds (up to ≥240 kph) scour some alpine ridges, keeping them snow-free during winter ([Bibr ref59][Bibr ref59][Bibr ref59]). During summer, Sierra Nevada bighorn sheep live in alpine habitats at moderate to high elevations (>3300 m). Some bighorn remain in alpine habitats at high elevations (sometimes >4000 m) during winter, but most live at more moderate elevations (1500–2700 m) ([Bibr ref60][Bibr ref60][Bibr ref60]). Mountain lions (*Puma concolor*) are the most common predator of adult bighorn in this system, which also prey on mule deer and can result in apparent competition ([Bibr ref65][Bibr ref65][Bibr ref65][Bibr ref65][Bibr ref65][Bibr ref65][Bibr ref65]; [Bibr ref35][Bibr ref35][Bibr ref35]; [Bibr ref28][Bibr ref28][Bibr ref28]).

### Animal capture, handling and monitoring

Animal research was conducted in accordance with US Fish and Wildlife Service Permit TE050122–6 and California Department of Fish and Wildlife Animal Welfare Policy (2018–02), and capture protocols (Sierra Nevada Bighorn Sheep Capture Plan 2006–10–2018-10) were approved annually. During 2006–18, we captured Sierra bighorn using a net gun fired from a helicopter. Animals were hobbled, blindfolded, bagged, slung from a helicopter and ferried to a staging area for processing and data collection. During handling of Sierra bighorn, we collected data on body mass and fat using manual palpation scores of body condition and ultrasound measurements of maximum depth of rump fat ([Bibr ref62][Bibr ref62][Bibr ref62]). We converted body condition scores and ultrasonic measurements of rump fat to estimate ingesta-free body fat (IFBFat) using equations validated for bighorn sheep ([Bibr ref62][Bibr ref62][Bibr ref62]) and then scaled these measurements following [Bibr ref20][Bibr ref20][Bibr ref20]. We also fit various models of GPS collars (various models manufactured by ATS, Isanti, MN; Lotek, Newmarket, Canada; North Star, Oakton, USA; Tellus, Lindesberg, Sweden; Telonics, Mesa, USA; Vectronic Aerospace, Berlin, Germany) programmed to collect 1–24 GPS locations per day on each Sierra bighorn.

### Classification of migratory behaviours

We used migrateR ([Bibr ref58][Bibr ref58][Bibr ref58]), to fit generalized linear models to elevation data in time series to classify migratory behaviours of Sierra bighorn. MigrateR extends net-squared displacement models often used for long-distance migration ([Bibr ref9][Bibr ref9][Bibr ref9]) to elevational migration ([Bibr ref58][Bibr ref58][Bibr ref58]) and subsampled all data to the first location recorded each day for determination of migratory behaviours. We did not include data from animals in the first year following translocation to a new range because these animals lacked knowledge possessed by migrants ([Bibr ref34][Bibr ref34][Bibr ref34]) and therefore these movements would have been more exploratory in nature, rather than an intentional, directional migration. Following the methods of [Bibr ref21][Bibr ref21][Bibr ref21], we expanded the migratory classifications of [Bibr ref58][Bibr ref58][Bibr ref58] to classify migratory tactics as traditional migration, residency, abbreviated migration and vacillating migration. Traditional migrants were those individuals that migrated between seasonal ranges that were spatially separated and whose elevational-time series data showed a distinct drop in elevation, a continuous period of occupancy on the secondary range and a return to higher elevations after the period of occupancy. Residents were animals whose elevational-time series data were best described by a linear model because these individuals remained at approximately the same elevation year-round. Abbreviated migrants wintered at high elevations, but then made a 2- to 3-week movement to lower elevations in late spring when snow has subsided at lower elevations and green-up commenced. Vacillating migrants made multiple round-trip movements between high- and low-elevation ranges during winter. Final determinations of migratory tactics were based on Akaike Information Criterion corrected for small sample size (AICc) ranking ([Bibr ref2][Bibr ref2][Bibr ref2][Bibr ref2]; [Bibr ref10][Bibr ref10][Bibr ref10][Bibr ref10]) of migratory models and visual inspections of plots ([Bibr ref45][Bibr ref45][Bibr ref45]; [Bibr ref58][Bibr ref58][Bibr ref58]; [Bibr ref21][Bibr ref21][Bibr ref21]). We also estimated linear distance moved between high- and low-elevation ranges for migratory animals using migrateR and used this information in our analyses.

### Statistical analyses

All statistical analyses were conducted in Stata 14 (StataCorp, College Station, TX), except for Chi-squared equality of proportions tests that we conducted in R ([Bibr ref50][Bibr ref50][Bibr ref50][Bibr ref50]). To evaluate how autumn body fat influenced migration, we used analysis of variance, multinomial logistic regression and linear regression. First, we used a Chi-squared equality of proportions test to evaluate whether composition of migratory tactics differed amongst populations (i.e. 14 individual herds of Sierra bighorn). We used RU, which essentially represents metapopulations whose habitats share similar characteristics, rather than herd in analyses with body fat owing to small sample sizes within populations. RU was used as a surrogate of availability of high-elevation and low-elevation winter ranges to account for the influence of the availability of different types of range on migratory propensity ([Bibr ref59][Bibr ref59][Bibr ref59]). High- and low-elevation habitats differ amongst RUs (along a north–south gradient) and have different risks in terms of nutrition, snow and predation that could influence migratory decisions. Additionally, reintroduction timelines differ across RUs, which could affect migratory propensity given the length of time required for migration to become re-established following reintroduction ([Bibr ref34][Bibr ref34][Bibr ref34]).

We used analysis of variance to evaluate differences in autumn body fat across migratory tactics (i.e. resident [including abbreviated migrants that are functionally most like residents in terms of winter risk], traditional migrant, vacillating migrant). We used multinomial logistic regression to evaluate main and interaction effects of autumn body fat and RU on probability of migrating (any migratory tactic) versus residency; we grouped residents and abbreviated migrants into one category owing to small sample sizes. The Kern RU was excluded from analyses incorporating RU as a covariate owing to small sample sizes for body fat (*n* = 1). We used linear regression to evaluate the influence of autumn body fat on distance migrated, including RU as a covariate and evaluated potential interaction terms. We also used linear regression to examine whether switching rates were a function of mean population-level body fat across all years of data because in-year sample sizes were too small. To determine the most supported model amongst candidate models in regression analyses we used an information-theoretic approach and ranked models using Akaike’s Information Criterion corrected for small sample size and examined models for uninformative parameters ([Bibr ref2][Bibr ref2][Bibr ref2][Bibr ref2]; [Bibr ref10][Bibr ref10][Bibr ref10][Bibr ref10]; Arnold,^2010^). Where we did not have *a priori* competing models to compare, we used frequentist statistics rather than an information-theoretic approach. For all tests of significance, we used α = 0.10, rather than α = 0.05, owing to small samples sizes and our greater tolerance for committing a Type II error than a Type I error. Our small sample sizes reduced statistical power for our analyses and therefore increased the likelihood that we would fail to reject a false null hypothesis and miss important biological effects based on an arbitrary α. Traditional use of α = 0.05 has been noted to be negatively impacted by small sample size and use of α > 0.05 has received increasing acceptance for complex relationships or small sample size as has α < 0.05 for large sample sizes, partly in efforts to avoid Lindley’s paradox ([Bibr ref64][Bibr ref64][Bibr ref64]; [Bibr ref40][Bibr ref40][Bibr ref40][Bibr ref40]). 

## Results

**Figure 1:**
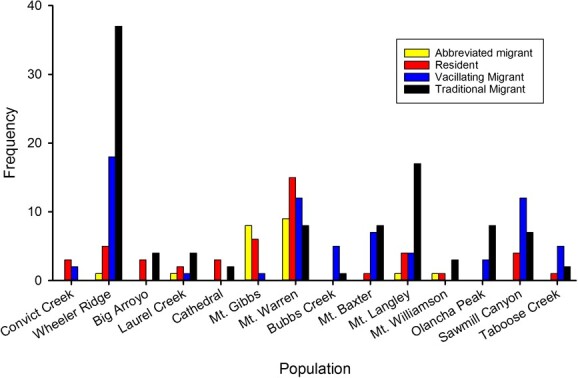
Migratory tactics of male Sierra Nevada bighorn sheep in *n* = 14 populations in the Central (Convict Creek, Wheeler Ridge), Kern (Big Arroyo, Laurel Creek), Northern (Cathedral, Mt. Gibbs, Mt. Warren) and Southern (Bubbs Creek, Mt. Baxter, Mt. Langley, Mt. Williamson, Olancha Peak, Sawmill Canyon, Taboose Creek) RUs California, USA, from 2006 to 2019.

We classified migratory behaviours for 240 animal-years for 129 individual male Sierra bighorn. We classified migratory behaviour for 60 animals in 1 year, 40 animals in 2 years, 19 animals in 3 years, seven animals in 4 years and for three animals in 5 years. Migration was the predominant behavioural state as 171 animal-years were classified as migrants, which included traditional migrants (*n* = 101) and vacillating migrants (*n* = 70). Residency was documented for 69 animal-years, which included *n* = 48 instances of residency alone and *n* = 21 instances of residency with abbreviated migration. Traditional migration, residency and abbreviated migration occurred in all RUs, but vacillating migration was not documented in the Kern RU. Proportions of traditional migrants, vacillating migrants, residents and abbreviated migrants differed amongst the four RUs (χ^2^_[9]_ = 73.12, *P* < 0.001; Fig. 1).

**Table 1:** Multinomial logistic models characterizing probability of male Sierra Nevada bighorn sheep choosing a migratory tactic (residency, traditional migration or vacillating migration; determined from classification of movement data in migrateR ([Bibr ref58][Bibr ref58][Bibr ref58])); and linear regression models characterizing the relationship between distance separating high- and low-elevation seasonal ranges of migratory male Sierra Nevada bighorn sheep relative to ingesta-free body fat in autumn (AutumnIFBFat) and Recovery Unit (RUCode; categorical variable with 3 levels (Central, Northern, Southern); Kern was excluded due to lack of Autumn IFBFat samples) in California, USA, from 2006 to 2019. *K* is the number of parameters, *n* is sample size, LL is log likelihod, AICc is Akaike Information Criterion corrected for small sample size, ΔAICc is the change in AICc from the top model, and ωi is the Akaike model weight

Response variable	Candidate Models	*K*	*n*	LL	AICc	ΔAICc	ωi
Probability of choosing residency, traditional migration or vacillating migration	AutumnIFBFat*RUCode	8	65	−61.834	142.239	0.000	0.860
	AutumnIFBFat + RUCode	8	65	−63.837	146.245	4.006	0.116
	AutumnIFBFat + RUCode + AutumnIFBFat*RUCode	12	65	−59.718	149.437	7.198	0.024
							
Distance moved (difference in elevation between seasonal ranges)	AutumnIFBFat + RUCode	4	47	−356.194	721.341	0.000	0.676
	AutumnIFBFat*RUCode	4	47	−357.197	723.346	2.005	0.248
	AutumnIFBFat + RUCode + AutumnIFBFat*RUCode	6	47	−355.799	725.699	4.358	0.076

**Figure 2:**
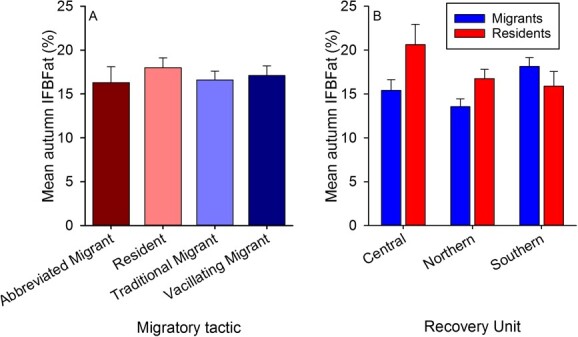
Mean (± SE) ingesta-free body fat percentage (IFBFat (%)) of male Sierra Nevada bighorn sheep in autumn by migratory tactic (*n* = 4 abbreviated migrant, 14 resident, 21 traditional migrant, 26 vacillating migrant) across *n* = 11 populations in the Central, Southern and Northern RUs combined (A) and mean (± SE) IFBFat (%) of combined migratory (traditional and vacillating migration) and resident (abbreviated migration and residency) tactics within the Central (*n* = 8, 5, migrants, residents), Northern (*n* = 8, 9) and Southern (*n* = 31, 4) RUs (B) in California, USA, from 2006 to 2019.

**Figure 3:**
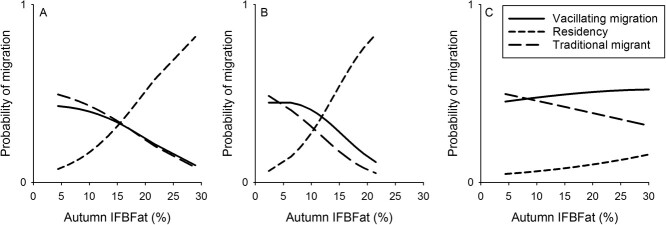
Relationship between autumn infesta-free body fat percentage (IFBFat (%)) in autumn of male Sierra Nevada bighorn sheep and predicted probability of migratory tactics for animals in the Central (A; *n* = 13), Northern (B; *n* = 17) and Southern (C; *n* = 35) RUs in California, USA, from 2006 to 2019.

On average, resident males had the greatest body fat levels in autumn, followed by vacillating migrants, traditional migrants and abbreviated migrants, but differences were not significant in analysis of variance (*P* = 0.860, df = 3, 61, F = 0.25) (Fig. 2A). Nevertheless, by RU, residents tended to have greater autumn IFBFat levels than migrants, except in the Southern RU (Fig. 2B). The top model for probability of migration included only an interaction term for body fat and RU (Table 2, Fig. 3). Probability of migration dropped off precipitously for animals in the Central and Northern RUs when autumn IFBFat ≥10% (Fig. 3). For every 1-percentage point increase in body fat, there was a 16% decrease in the likelihood that a Sierra bighorn in the Central RU would be a vacillating migrant, compared with a resident and a 21% decrease for Sierra bighorn in the Northern RU (Fig. 3, Table 2). Similarly, for every 1-percentage point increase in body fat there was a 17% decrease in the likelihood that an animal would be a traditional migrant in the Central RU and a 25% decrease in the Northern RU (Fig. 3, Table 2). There was no relationship between body fat and probability of migration for animals in the Southern RU (Table 4).

**Table 2:** Results from top multinomial logistic models, as indicated by Akaike Information Criteria corrected for small sample size (Table 1), characterizing probability of male Sierra Nevada bighorn sheep choosing a migratory tactic (traditional migration or vacillating migration) relative to residency as a function of AutumnIFBFat and RU (RUCode; categorical variable with 3 levels (Central, Northern, Southern); Kern excluded due to lack of Autumn IFBFat samples) in California, USA, from 2006 to 2018

Parameter	Coef.	SE	t	*P*
Vacillating migrants				
AutumnIFBFat*Central	−0.16	0.09	−1.75	0.08
AutumnIFBFat*Northern	−0.21	0.10	−1.99	0.05
AutumnIFBFat*Southern	−0.04	0.08	−0.52	0.60
				
Intercept	2.43	1.49	1.63	0.10
				
Traditional migrants				
AutumnIFBFat*Central	−0.17	0.09	−1.81	0.07
AutumnIFBFat*Northern	−0.25	0.11	−2.26	0.02
AutumnIFBFat*Southern	−0.06	0.08	−0.78	0.44
				
Intercept	2.62	1.54	1.71	0.09

**Figure 4:**
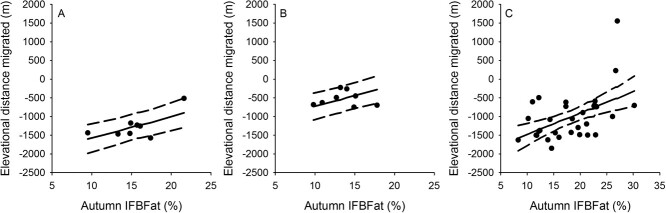
Relationship between ingesta-free body fat percentage (IFBFat (%)) in autumn of male Sierra Nevada bighorn sheep and elevational distance migrated (shown as predicted values (solid line) with 95% confidence intervals as dashed lines) for animals in the Central (A; *n* = 8), Northern (B; *n* = 8) and Southern (C; *n* = 31) RUs in California, USA, from 2006 to 2019.

Sierra bighorn in the Central and Southern RUs moved further down in elevation than in the Northern RU (Table 3, Fig. 4). Distances separating high- and low-elevation ranges averaged 1176 ± 176 m for Sierra bighorn in the Central RU, 331 ± 182 m in the Northern RU and 1087 ± 91 in the Southern RU (marginal mean ± SE). Body fat influenced fine-scale migratory behaviours. Amongst vacillating and traditional migrants, body fat influenced the distance between migratory and resident ranges (Fig. 4, Table 3). For every 1-percentage point increase in autumn body fat, the difference between high- and low-elevation ranges decreased by ~51 m, on average, for Sierra bighorn and differed amongst populations (Fig. 4, Table 3).

**Table 3:** Results from top linear regression models, as indicated by Akaike Information Criteria corrected for small sample size (Table 1), characterizing the relationship between distance separating high- and low-elevation seasonal ranges of migratory Sierra Nevada bighorn sheep relative to ingesta-free body fat percentage in autumn (autumnIFBFat) and recovery unit (RU; categorical variable with 3 levels (Central, Northern, Southern); Kern excluded due to lack of Autumn IFBFat samples) in California, USA, from 2006 to 2019

Model Parameter	Coef.	SE	t	*P*
AutumnIFBFat	51.0	13.4	3.80	<0.001
				
RU Northern	845	249	3.40	0.001
RU Southern	90	201	0.45	0.658
				
Intercept	−2089	279	−7.48	<0.001

**Figure 5:**
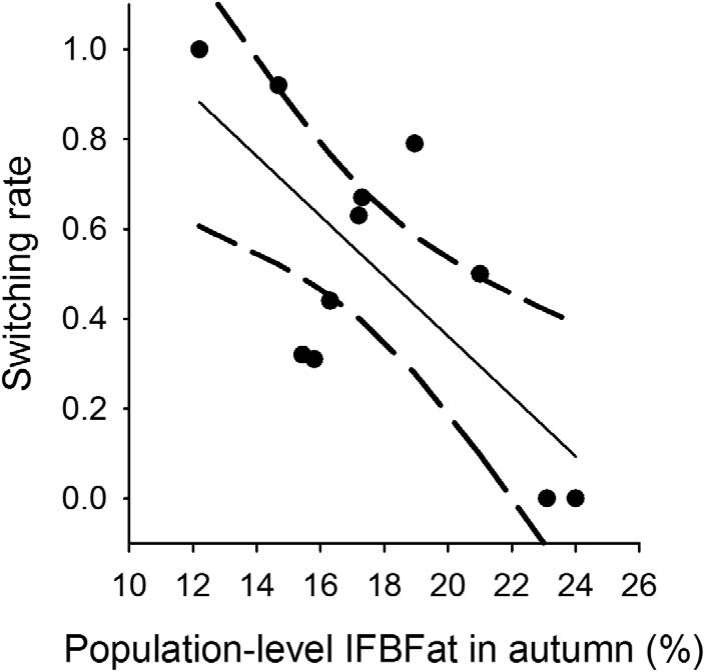
Relationship between population-level IFBFat (%) in autumn of male Sierra Nevada bighorn sheep and switching rate (shown as predicted values (solid line) with 95% confidence intervals as dashed lines) where each circle represents one population in California, USA, from 2006 to 2019.

Male Sierra bighorn switched migratory tactics between years 60 times out of 111 opportunities to switch, for a switching rate of 54%. Males switched from migratory to residency behaviours *n* = 19 times (32% of switches) and from residency to migratory behaviours *n* = 7 times (12%). The remaining switches were from one migratory tactic to another or one residency tactic to another: vacillating migrant to traditional migrant (*n* = 13; 22%); traditional migrant to vacillating migrant (*n* = 9; 16%); abbreviated migrant to resident (*n* = 9; 15%); resident to abbreviated migrant (*n* = 3; 5%). Amongst 30 individuals tracked over ≥3 years, *n* = 14 switched tactics more than once, including *n* = 13 individuals that switched tactics twice, two individuals that switched tactics three times and one individual that switched tactics four times. Additionally, body fat influenced switching rates at the population level (*P* = 0.012, R^2^ = 0.521, adj. R^2^ = 0.468, *n* = 11; Table 4), with switching rate decreasing 7 percentage points for every 1 percentage point increase in IFBFat (Fig. 5).

**Table 4:** Results from a linear regression model characterizing the relationship between population-level ingesta-free body fat (IFBFat) in autumn and population-level switching rates for *n* = 11 populations of Sierra Nevada bighorn sheep in California, USA, from 2006 to 2019

Parameter	Coef.	SE	t	*P*
AutumnIFBFat	−0.07	0.02	−3.13	0.012
Intercept	1.70	0.39	4.38	0.002

## Discussion

Body fat is an important adaptation to seasonal environments through its direct influence on reproduction and survival ([Bibr ref17][Bibr ref17][Bibr ref17]; [Bibr ref44][Bibr ref44][Bibr ref44]; [Bibr ref62][Bibr ref62][Bibr ref62]; [Bibr ref19][Bibr ref19][Bibr ref19]). We hypothesized that the importance of body fat to animals living in seasonal environments would extend beyond direct effects by influencing migratory behaviours in a risk-sensitive manner. In our study area, residents remain at high elevations year-round, which means they experience harsh winter conditions and rely more on body fat for survival than migrants that winter at low elevations ([Bibr ref19][Bibr ref19][Bibr ref19]); migrants however incur increased predation risk ([Bibr ref36][Bibr ref36][Bibr ref36]; [Bibr ref28][Bibr ref28][Bibr ref28]). Because of the contrasting risks associated with choice of migratory tactic and migration distance, we predicted that probability of migration would be inversely related to body fat as would migration distance. The results supported both predictions: probability of migration and migration distance were inversely related to body fat such that skinnier animals were more likely to migrate to lower elevation winter ranges and migration distance increased with decreasing body fat. Rates of switching migratory tactics across years increased as body fat decreased, reiterating that body fat acts as a buffer and ameliorates the needs of animals to be wholly responsive to their immediate environment. Collectively, our findings support the hypothesis that migration was risk-sensitive to bodyfat.

Our study adds to a growing body of work that relates body fat with migratory behaviours, including timing or initiation of migration ([Bibr ref43][Bibr ref43][Bibr ref43]; [Bibr ref54][Bibr ref54][Bibr ref54]), as a fuel for migration ([Bibr ref49][Bibr ref49][Bibr ref49][Bibr ref49]) or as a cue for departing stopover sites ([Bibr ref29][Bibr ref29][Bibr ref29]). In our study, migratory behaviours were a risk-sensitive function of body fat, particularly where the relative value of body fat as a nutritional buffer varies across behavioural states ([Bibr ref19][Bibr ref19][Bibr ref19]). For example, animals with smaller body fat stores were more likely to migrate to low-elevation winter ranges likely because starvation is imminent at high elevations during winter without adequate fat reserves ([Bibr ref19][Bibr ref19][Bibr ref19]). High levels of body fat are a requisite nutritional buffer for high-elevation residents to persist in environments characterized by extreme winters ([Bibr ref65][Bibr ref65][Bibr ref65][Bibr ref65][Bibr ref65][Bibr ref65][Bibr ref65]; Spitz,^2015^; [Bibr ref15][Bibr ref15][Bibr ref15]) that limit food availability and increase energy demands for locomotion and thermoregulation ([Bibr ref13][Bibr ref13][Bibr ref13][Bibr ref13]; [Bibr ref24][Bibr ref24][Bibr ref24][Bibr ref24]). In the Sierra Nevada, high-elevation residents require almost twice as much body fat as migrants to achieve >90% overwinter survival ([Bibr ref19][Bibr ref19][Bibr ref19]). Smaller body fat stores of migrants likely precluded them from pursuing residency because they lacked an adequate nutritional buffer against harsh winter conditions at high elevations. Instead, small fat stores may have forced animals to seek milder conditions at lower elevations where they would have more predictable access to food, but at the cost of predation risk ([Bibr ref36][Bibr ref36][Bibr ref36]; Spitz,^2015^; [Bibr ref28][Bibr ref28][Bibr ref28]). Thus, migrants chose a migratory tactic that was risk-averse to starvation, but risk-prone to predation, in contrast with residents that were risk-averse to predation and risk-prone to starvation.

The elevational distance an animal migrated also was risk-sensitive to body fat. As expected, animals with less body fat migrated further down in elevation than animals with more body fat, presumably because as their nutritional buffer declined animals would have to move further down in elevation to escape snow and access food supplies. Choosing migration distance relative to body fat stores likely increased the relative value of the nutritional buffer provided by their fat stores; for example a given amount of body fat should last longer in an environment with little snow, where food is more abundant and energy expenditures are lower, compared to one where snow limits food availability and increases locomotory costs ([Bibr ref19][Bibr ref19][Bibr ref19]). Choosing a migration distance relative to body fat stores likely enabled Sierra bighorn to attune their risk relative to the buffer provided by different levels of body fat without exposing themselves to unnecessary risk of predation. That is, Sierra bighorn could have chosen to migrate further to increase access to food, but they did not need to because of the nutritional buffer afforded by their body fat stores. Thus, attuning migration distance to body fat stores may have allowed Sierra bighorn to balance trade-offs between starvation and predation.

Our study focused exclusively on migratory behaviours of males, which was atypical for studies of ungulate migration (Supplementary Information 1; Supplementary Table S1). Focusing on males allowed us to explore relations between physiological state and migration without the potentially confounding influence of having a lamb at heel (Festa-Bianchet,^1988^) and allowed us to examine potential differences in migratory behaviour between sexes that have different annual physiological cycles. Elevational distance migrated by males did not differ appreciably from those reported for females ([Bibr ref60][Bibr ref60][Bibr ref60]), but males switched tactics more frequently ([Bibr ref60][Bibr ref60][Bibr ref60]). Higher switching rates of males versus females may reflect greater behavioural flexibility given their lack of parental investment in offspring and have been reported in other partially migratory ungulates ([Bibr ref48][Bibr ref48][Bibr ref48]). Whereas males may be able to choose a migratory tactic based solely on their needs, females with a lamb at heel must also consider the needs of the lamb. Although most female Sierra bighorn do not lactate over winter, they retain strong bonds with their offspring until they prepare to give birth to subsequent offspring in May, and given that the first year of life is one of greatest vulnerability in ungulates ([Bibr ref27][Bibr ref27][Bibr ref27]), mothers should enhance their fitness by supporting offspring through the first year. Keeping lambs away from predators by remaining at high elevations can reduce predation risk to lambs, but lambs invest in growth rather than accretion of body fat in their first summer and may be more susceptible to mortality from starvation if wintering at higher elevations. Thus, females may be more inclined to migrate regardless of body fat if winter conditions on high-elevation ranges increase risk of mortality for lambs. However, females below a body fat threshold of ~14% may be forced to migrate ([Bibr ref19][Bibr ref19][Bibr ref19]) to survive, which also may contribute to their lower migratory plasticity compared to males. Greater migratory plasticity of males versus females may be an important consideration for re-establishing desired migrations.

In addition to individual-level effects of body fat on migratory behaviours, body fat also was related to migratory plasticity (i.e. switching rates). At the population level, switching became more common with decreasing body fat levels, in accordance with our hypothesis that body fat underpins facultative migration—an observation consistent in birds ([Bibr ref18][Bibr ref18][Bibr ref18]). In the Sierra Nevada, decreasing body fat levels are associated with lower levels of survival, especially for residents ([Bibr ref19][Bibr ref19][Bibr ref19]). Thus, as body fat levels decrease, animals exhibiting migratory plasticity should modulate their risk of starvation by switching migratory tactics. At the population level, fluctuating levels of body fat also could lead to changes in the composition of migratory tactics within the population. Not all taxa exhibit high levels of migratory plasticity and in less plastic species, such as mule deer (*Odocoileus hemionus hemionus*; [Bibr ref52][Bibr ref52][Bibr ref52]), the inability to be flexible may mean that they cannot modulate the value of body fat through migration and therefore must achieve greater levels of body fat prior to winter. Additionally, greater variation in body fat levels in less plastic species may result in more variable survival given the relationship between body fat and survival ([Bibr ref19][Bibr ref19][Bibr ref19]). In the absence of additional data, we caution interpretation of this finding until additional data can substantiate (or refute) it. Nevertheless, population-level effects of body fat and migratory tactic may be imminent, especially given that the nutritional buffer provided by body fat can buffer vital rates of populations from severe weather ([Bibr ref33][Bibr ref33][Bibr ref33]). Differences in body fat across migratory tactics andpopulations also may help explain why vital rates of migrants and residents often differ ([Bibr ref46][Bibr ref46][Bibr ref46]; [Bibr ref42][Bibr ref42][Bibr ref42]; [Bibr ref66][Bibr ref66][Bibr ref66]; [Bibr ref31][Bibr ref31][Bibr ref31]; [Bibr ref19][Bibr ref19][Bibr ref19]).

Although our results consistently supported our hypotheses, several important caveats are present. Our sample size for males was relatively large for published studies of ungulate migration (Supplementary Information 1;Supplementary Table S1) and body fat, but small within RU and for switching rates for periods >2 years. Because of small sample sizes, we relaxed confidence levels and accepted a higher risk of a Type II error than a Type I error. Despite these limitations, consistency in our results across migratory behaviours at individual and population levels strongly support our hypothesis that migratory behaviours were risk-sensitive to body fat. Further, our findings can be explained mechanistically, which also bolsters our confidence that migration was risk-sensitive to body fat, with residents being risk-prone to starvation and migrants being risk-averse.

Our study provided a snapshot into how animals modulate risk in seasonal environments via interactions between behaviour and physiology. For bighorn sheep in the Sierra Nevada, males chose between high-elevation residency during winter where they are heavily reliant on stored fat reserves to survive a food-limited winter, migration to low-elevation ranges where food is more abundant and fat reserves less necessary for survival but risk of predation is higher or an intermediate tactic where they switched between residency and migration presumably offering flexibility in response to both fat reserves and environmental conditions ([Bibr ref21][Bibr ref21][Bibr ref21]). Body fat varies across populations ([Bibr ref62][Bibr ref62][Bibr ref62]) and not all animals in this system can achieve high levels of body fat over summer, which suggests some degree of nutritional limitation of summer range ([Bibr ref17][Bibr ref17][Bibr ref17]; [Bibr ref44][Bibr ref44][Bibr ref44]; [Bibr ref55][Bibr ref55][Bibr ref55]; [Bibr ref19][Bibr ref19][Bibr ref19]). Nonetheless, animals should take advantage of environmental complexity to modulate risk relative to the insurance provided by their body fat. Sierra bighorn with high levels of body fat had insurance against starvation, but body fat also provided indirect insurance against predation in two ways. First, predation risk is very low for high-elevation residents, so being fat enough to persist at high elevation through winter indirectly reduced predation risk. Second, the greater body fat an animal had, the shorter elevational distance it had to migrate, which would reduce their seasonal sympatry with predators. Thus, the consequences of not fattening may be stark in that skinny animals generally had to trade off an imminent risk (starvation) for a consistent reward (food at lower elevations); a finding consistent with the risk-sensitivity hypothesis ([Bibr ref12][Bibr ref12][Bibr ref12]). The food reward at lower elevation, however, came with a less predictable risk (predation). Predator–prey dynamics and other ecosystem-level phenomena that are influenced by risk-sensitive migration, however, could change as a function of climate-related shifts in the relative risks associated with each migratory tactic. Nevertheless, our findings suggest that risk-sensitive migration behaviour yields a layer of flexibility for migratory ungulates that should aid in their long-term persistence and may be critical to their recovery. Understanding relations between behaviour and physiology may aid conservation practitioners in making decisions about recovery planning for species in complex environments. In particular, conservation efforts to support persistence of diverse migratory portfolios and high-quality summer ranges that support fat accretion may allow animals the necessary flexibility to modulate risk by attuning behaviour to physiological state and could yield desirable conservation outcomes.

## Supplementary Material

Web_Material_coae029
