# Supplementary material for: Migratory behaviours are risk-sensitive to physiological state in an elevational migrant
Source: Conserv Physiol. 2024 May 17;12(1):coae029. doi: 10.1093/conphys/coae029 (PMC11109817; doi:10.1093/conphys/coae029)
Supplement: Web_Material_coae029 [file web_material_coae029.zip › Supplementary_Information_1 (1).pdf]

Supplementary Information 1. Denryter, K., T.R. Stephenson, and K.L. Monteith. Migratory behaviors are risk sensitive to physiological state in an elevational migrant. 2024. *Conservation Physiology*.

Table S1. Comparison of selected studies of ungulate migration demonstrating a sampling bias toward females.

Common Name	Scientific Name	*n* Males	*n* Females	Source
Caribou	*Rangifer tarandus*	14	40	(Mahoney & Schaefer, 2002)
Chamois	*Rupicapra rupicapra*	21	0	(Lovari, Sacconi, & Trivellini, 2010)
Chamois	*Rupicapra rupicapra*	12	9	(Unterthiner, Ferretti, Rossi, & Lovari, 2012)
Chamois	*Rupicapra rupicapra*	.	.	(Clarke, 1986)
Elk	*Cervus elaphus*	12	38	(Paton, Ciuti, Quinn, & Boyce, 2017)
European bison	*Bison bonasus*	.	.	(Kowalczyk, 2013)
Himalayan tahr	*Hemitragus jemlahicus*	.	.	(Forsyth, 1999)
Impala	*Aepyceros melampus*	64	272	(Gaidet & Lecomte, 2013)
Moose	*Alces alces*	64	334	(Singh, Börger, Dettki, Bunnefeld, & Ericsson, 2012)
Moose	*Alces alces*	80	11	(Bunnefeld et al., 2011)
Mountain goats	*Oreamnos americanus*	11	11	(K. S. White, 2006)
Mountain goats	*Oreamnos americanus*	11	31	(Rice, 2008)
Mule deer	*Odocoileus hemionus*	12	27	(Brown, 1992)
Mule deer	*Odocoileus hemionus*	7	22	(Nicholson, Terry Bowyer, & Kie, 1997)
Pronghorn	*Antilocapra americana*	20*	40*	(Kolar, Millspaugh, & Stillings, 2011)
Pronghorn	*Antilocapra americana*	0	44	(P. J. White, Davis, Barnowe-Meyer, Crabtree, & Garrott, 2007)
Red deer	*Cervus elaphus*	60	168	(Rivrud et al., 2016)
Red deer	*Cervus elaphus*	20	0	(Kropil, Smolko, & Garaj, 2015)
Red deer	*Cervus elaphus*	96	0	(Jarnemo, 2008)
Red deer	*Cervus elaphus*	73	221	(Bischof et al., 2012)
Red deer	*Cervus elaphus*	12	37	(Mysterud, Qviller, Meisingset, & Viljugrein, 2016)
Reindeer	*Rangifer tarandus*	1	8	(Tyler & Øritsland, 1989)

Roe deer	*Capreolus capreolus*	10	13	(Mysterud, 1999)
Roe deer	*Capreolus capreolus*	.	.	(Cagnacci et al., 2011)
Sika deer	*Cervus nippon*	6	17	(Takii, Izumiyama, & Taguchi, 2012)
White-tailed deer	*Odocoileus virginianus*	6	14	(Luccarini, Mauri, Ciuti, Lamberti, & Apollonio, 2010)
White-tailed deer	*Odocoileus virginianus*	0	39	(Nixon et al., 2008)
Wildebeest	*Connochaetes taurinus*	.	.	(Morrison & Bolger, 2012)

References

Bischof, R., Loe, L. E., Meisingset, E. L., Zimmermann, B., Van Moorter, B., & Mysterud, A. (2012). A migratory northern ungulate in the pursuit of spring: jumping or surfing the green wave? *The American Naturalist*, *180*, 407–424.

Brown, C. G. (1992). Movement and migration patterns of mule deer in southeastern Idaho. *Journal of Wildlife Management*, *56*, 246–253.

Bunnefeld, N., Börger, L., Van Moorter, B., Rolandsen, C. M., Dettki, H., Solberg, E. J., & Ericsson, G. (2011). A model-driven approach to quantify migration patterns: individual, regional and yearly differences. *Journal of Animal Ecology*, *80*, 466–476.

Cagnacci, F., Focardi, S., Heurich, M., Stache, A., Hewison, A. J. M., Morellet, N., … Urbano, F. (2011). Partial migration in roe deer: migratory and resident tactics are end points of a behavioural gradient determined by ecological factors. *Oikos*, *120*, 1790–1802.

Clarke, C. M. H. (1986). Chamois movements and habitat use in the Avoca River area, Canterbury, New Zealand. *New Zealand Journal of Zoology*, *13*, 175–198.

Forsyth, D. M. (1999). Long-term harvesting and male migration in a New Zealand population of Himalayan tahr *Hemitragus jemlahicus*. *Journal of Applied Ecology*, *36*, 351–362.

Gaidet, N., & Lecomte, P. (2013). Benefits of migration in a partially-migratory tropical ungulate. *BMC Ecology*, *13*, 36.

Jarnemo, A. (2008). Seasonal migration of male red deer (Cervus elaphus) in southern Sweden and consequences for management. *European Journal of Wildlife Research*, *54*, 327–333.

Kolar, J. L., Millspaugh, J. J., & Stillings, B. A. (2011). Migration patterns of pronghorn in southwestern North Dakota. *Journal of Wildlife Management*, *75*, 198–203.

Kowalczyk, R. (2013). Movements of European bison (*Bison bonasus*) beyond the Białowieża Forest (NE Poland): range expansion or partial migrations? *Acta Theriologica*, *58*, 391–401.

Kropil, R., Smolko, P., & Garaj, P. (2015). Home range and migration patterns of male red deer Cervus elaphus in Western Carpathians. *European Journal of Wildlife Research*, *61*, 63–72.

Lovari, S., Sacconi, F., & Trivellini, G. (2010). Do alternative strategies of space use occur in male Alpine chamois? *Ethology Ecology and Evolution*, *18*, 221–231.

Luccarini, S., Mauri, L., Ciuti, S., Lamberti, P., & Apollonio, M. (2010). Red deer (*Cervus elaphus*) spatial use in the Italian Alps: home range patterns, seasonal migrations, and effects of snow and winter feeding. *Ethology Ecology and Evolution*, *18*, 127–145.

Mahoney, S. P., & Schaefer, J. A. (2002). Long-term changes in demography and migration of Newfoundland caribou. *Journal of Mammalogy*, *83*, 957–963.

Morrison, T. A., & Bolger, D. T. (2012). Wet season range fidelity in a tropical migratory ungulate. *Journal of Animal Ecology*, *81*, 543–552.

Mysterud, A. (1999). Seasonal migration pattern and home range of roe deer (*Capreolus capreolus*) in an altitudinal gradient in southern Norway. *Journal of the Zoological Society of London*, *247*, 479–486.

Mysterud, A., Qviller, L., Meisingset, E. L., & Viljugrein, H. (2016). Parasite load and seasonal migration in red deer. *Oecologia*, *180*(2), 401–407. doi: 10.1007/s00442-015-3465-5

Nicholson, M. C., Terry Bowyer, R., & Kie, J. G. (1997). Habitat selection and survival of mule

deer: tradeoffs associated with migration. *Journal of Mammalogy*, *78*, 483–504.

Nixon, C. M., Mankin, P. C., Etter, D. R., Hansen, L. P., Brewer, P. A., Chelsvig, J. E., … Sullivan, J. B. (2008). Migration behavior among female white-tailed deer in central and northern Illinois. *American Midland Naturalist*, *160*, 178–190.

Paton, D. G., Ciuti, S., Quinn, M., & Boyce, M. S. (2017). Hunting exacerbates the response to human disturbance in large herbivores while migrating through a road network. *Ecosphere*, *8*, e01841.

Rice, C. G. (2008). Seasonal altitudinal movements of mountain goats. *Journal of Wildlife Management*, *72*, 1706–1716.

Rivrud, I. M., Bischof, R., Meisingset, E. L., Zimmermann, B., Loe, L. E., Mysterud, & Mysterud, A. (2016). Leave before it's too late: anthropogenic and environmental triggers of autumn migration in a hunted ungulate population. *Ecology*, *97*, 1058–1068.

Singh, N. J., Börger, L., Dettki, H., Bunnefeld, N., & Ericsson, G. (2012). From migration to nomadism: movement variability in a northern ungulate across its latitudinal range. *Ecological Applications*, *22*, 2007–2020.

Takii, A., Izumiyama, S., & Taguchi, M. (2012). Partial migration and effects of climate on migratory movements of sika deer in Kirigamine Highland, central Japan. *Mammal Study*, *37*, 331–340.

Tyler, N. J. C., & Øritsland, N. A. (1989). Why don't Svalbard reindeer migrate? *Holarctic Ecology*, *12*, 369–376.

Unterthiner, S., Ferretti, F., Rossi, L., & Lovari, S. (2012). Sexual and seasonal differences of space use in Alpine chamois. *Ethology Ecology and Evolution*, *24*, 257–274.

White, K. S. (2006). Seasonal and sex-specific variation in terrain use and movement patterns of

mountain goats in southeastern Alaska. *Bienn. Symposium. North Wild Sheep and Goat Council*, *15*, 183–194.

White, P. J., Davis, T. L., Barnowe-Meyer, K. K., Crabtree, R. L., & Garrott, R. A. (2007). Partial migration and philopatry of Yellowstone pronghorn. *Biological Conservation*, *135*, 502–510.
