# Supplementary figures and images for: Migratory behaviours are risk-sensitive to physiological state in an elevational migrant
Source: Conserv Physiol. 2024 May 17;12(1):coae029. doi: 10.1093/conphys/coae029 (PMC11109817; doi:10.1093/conphys/coae029)

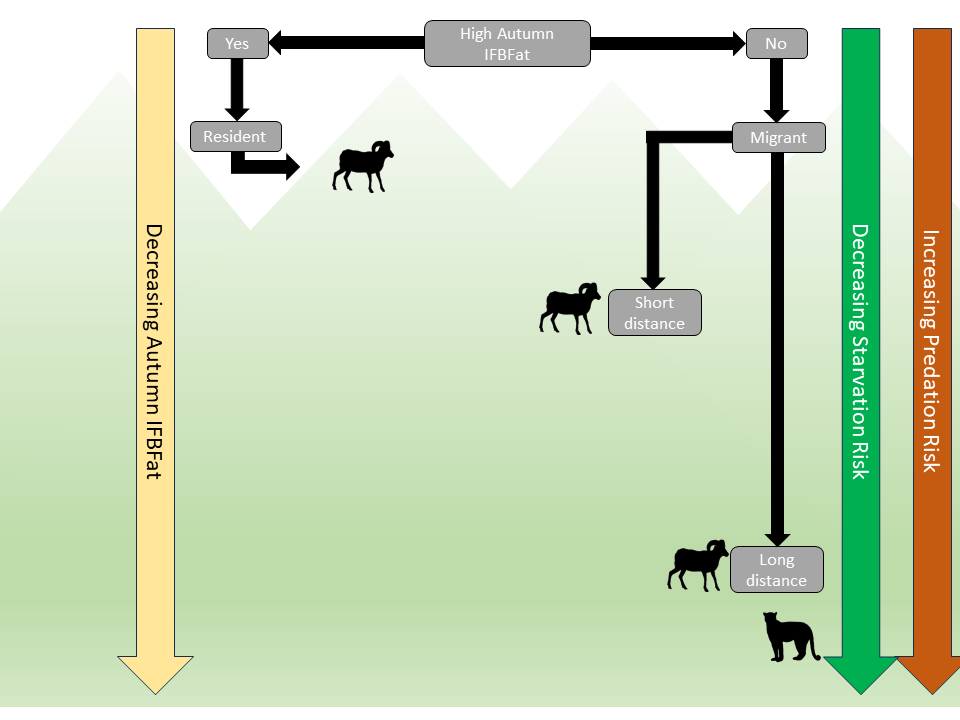

Supplement: Web_Material_coae029 [file web_material_coae029.zip › Graphical_Abstract (1).tif]
